# Vascular pedicle dissection time in laparoscopic colectomies as a novel marker of surgical skill: a prospective cohort study

**DOI:** 10.1007/s10151-025-03121-7

**Published:** 2025-03-22

**Authors:** Kirsten de Burlet, Isaac Tranter-Entwistle, Jeffrey Tan, Anthony Lin, Siraj Rajaratnam, Saxon Connor, Timothy Eglinton

**Affiliations:** 1 Department of General Surgery, Te Whatu Ora – Health New Zealand Waitaha, 124 Shakespeare Road, Takapuna, Auckland, 0620 New Zealand; 2 Department of General Surgery, Te Whatu Ora – Health New Zealand Waitaha, Wellington, New Zealand; 3 Department of General Surgery, Te Whatu Ora – Health New Zealand Waitaha, North Shore, Auckland, New Zealand

**Keywords:** Surgical skills, Colorectal surgery, Laparoscopic surgery, Textbook outcome, Competency assessment tool

## Abstract

**Background:**

Outcomes after colorectal resections depend on patient, pathology and operative factors. Existing validated surgical skills scores (such as the competency assessment tool (CAT)) are directly correlated with outcomes but are time-consuming to administer, limiting their clinical utility. The vascular pedicle dissection time (VPDT) is a novel, simple surgical skill assessment measure with the potential for computer vision automation. This study aimed to assess the VPDT and benchmark it against the CAT score.

**Methods:**

A prospective multicentre study was performed in New Zealand, recording videos of laparoscopic colorectal resections. Patient, operation and histology characteristics were also collected. The VPDT was calculated from retraction of the vascular pedicle to completion of medial dissection, including vascular division. Each laparoscopic video was scored by two independent colorectal surgeons, and the median CAT score was grouped into tertiles.

**Results:**

In total, 154 patients were included between December 2020 and November 2023 (74 (48.1%) right-sided and 80 (51.9%) left-sided resections). Median VPDT was significantly different between the CAT score groups for the right-sided resections (lower, 15 min; middle, 13 min; higher, 10 min; *p* = 0.036) and the left-sided resections (lower, 46 min; middle, 40 min; higher, 26 min; *p* =  < 0.001). There was no significant difference in R1 resection, anastomotic leak rate, the occurrence of Clavien–Dindo > 3 complications or re-admission between the CAT groups.

**Conclusions:**

This study showed that the VPDT was inversely correlated with the CAT score, indicating that it quantifies operative technical skill. A current study is evaluating the suitability of VPDT for real-time measurement using computer vision algorithms. This could allow for automated assessment of surgeons’ learning curve and skills.

## Introduction[No corrections]

Colorectal cancer is an important global health problem with significant morbidity and mortality [1]. New Zealand has one of the highest global incidences of colorectal cancer [2]. Surgical management remains the preferred treatment option for these patients, despite progress with medical alternatives [3–5]. Outcomes after surgery are dependent on patient, tumour and operative factors [6–10]. Recent studies have shown that operation duration and operative skill scores are directly correlated with adverse outcomes and, with that, reduced overall survival [10–14]. These studies have highlighted the utility of operative video recordings for skill assessment, training and audit.

Globally, general surgery training is transitioning from a focus on time and operative procedure numbers to competency-based training [15, 16]. Operative video review has the potential to be an important tool in competency-based training. Moreover, the development of platforms designed for this, such as Touch Surgery™, have facilitated its use. Despite this, manual review of operative videos remains time consuming, hence the emerging goal of identifying measures of operative competency suitable for automation using artificial intelligence techniques.

To date, artificial intelligence, in particular computer vision, has been applied to a small number of general surgical procedures, such as laparoscopic cholecystectomy [17], but has had limited application in colorectal surgery [18, 19]. The technology currently allows for phase segmentation of operations, has the potential for real-time anatomic identification [20] and has the abovementioned surgical skill feedback along with the development of appropriate metrics. One means of applying this technology is by quantifying key portions of the operation, such as the time it takes to perform the medial dissection around the vascular pedicle (vascular pedicle dissection time (VPDT)).

The aim of this study is to measure the VPDT in laparoscopic colectomies and to use this time as a benchmark to assess operative technical skill.

## Materials and methods

A multicentre prospective study was undertaken in three tertiary centres in New Zealand: Christchurch Hospital, North Shore Hospital and Wellington Hospital. Video recordings were collected for all laparoscopic colonic resections. Videos were analysed by two individual authors blinded to operator and patient background information. The competency assessment tool (CAT), a validated assessment tool for right hemicolectomies and anterior resections [12, 13], was used to quantify surgeon operative technical skill. The CAT score assesses four different parts of the operation: exposure, vascular pedicle dissection, mobilisation and anastomosis. Within each part, four further subcategories are captured. Each of these 16 assessment points are scored on a scale of 1–4, with 1 being dangerous or inadequate and 4 being excellent. In this study, we used a modified CAT score, as not all anastomoses were performed laparoscopically, making the minimum total score 12 and the maximum score 36. Where the CAT score differed more than three points between the two authors assessing the videos, a third author assessed and scored the video and a median score between all assessors was used as the final CAT score.

From the videos and operative records, total operative time and VPDT time was obtained. The vascular pedicle for right hemicolectomies is the ileocolic artery and for anterior resections the inferior mesenteric vein (IMV) and inferior mesenteric artery (IMA), separately.

### Definitions

Total operative time is defined as the time between knife to skin and completed skin closure. The VPDT is defined as the time taken from retraction of the vascular pedicle until completing the medial dissection and proceeding to the next step of the operation. Ligation time is defined as the time taken from retraction to ligation of the pedicle.

### Data collection and analysis

Data were collected from electronic records for patient characteristics, operation details, postoperative recovery and histology results. Clavien–Dindo > 3 complications are recorded in the electronic operation records. VPDT and CAT outcomes per operation were divided into tertiles, creating three groups (lowest, middle and highest). Outcomes, including length of stay (LOS), incidence of Calvien–Dindo(CD) > 3 [21] complication and oncologic results (resection margin and lymph node yield) were compared between the three groups. Data were analysed using SPSS (version 29, IBM). Median and interquartile range (IQR) were used for non-parametric continuous data and number and percentages for discrete data. Medians were compared using the Kruskal–Wallis test. Categorical data were compared using the chi-squared test. A multiple regression analysis was performed using the CAT score as the dependent variable and using the different patient, pathology and operation characteristics as independent variables. A *p* value of < 0.05 was considered statistically significant. The outcomes of the CAT scores from the individual assessing authors were compared using Kendall’s tau correlation test (for non-parametric, ranked and continuous data) [22].

## Cohort size

Statistical power was calculated using the RStudio “pwr” package pwr.f2.test function. With 13 independent coefficients, an estimated effect size of f^2^ = 0.10 (small–medium), an alpha level of 0.05 and power of 0.80, it was calculated that a sample size of 150 was needed to be adequately powered for the primary outcome.

### Ethics

Ethical approval was sought and granted from the Health and Disability Ethics Committee in New Zealand. Additional ethical approval was requested and granted from each participating institution.

## Results

**Fig. 1:**
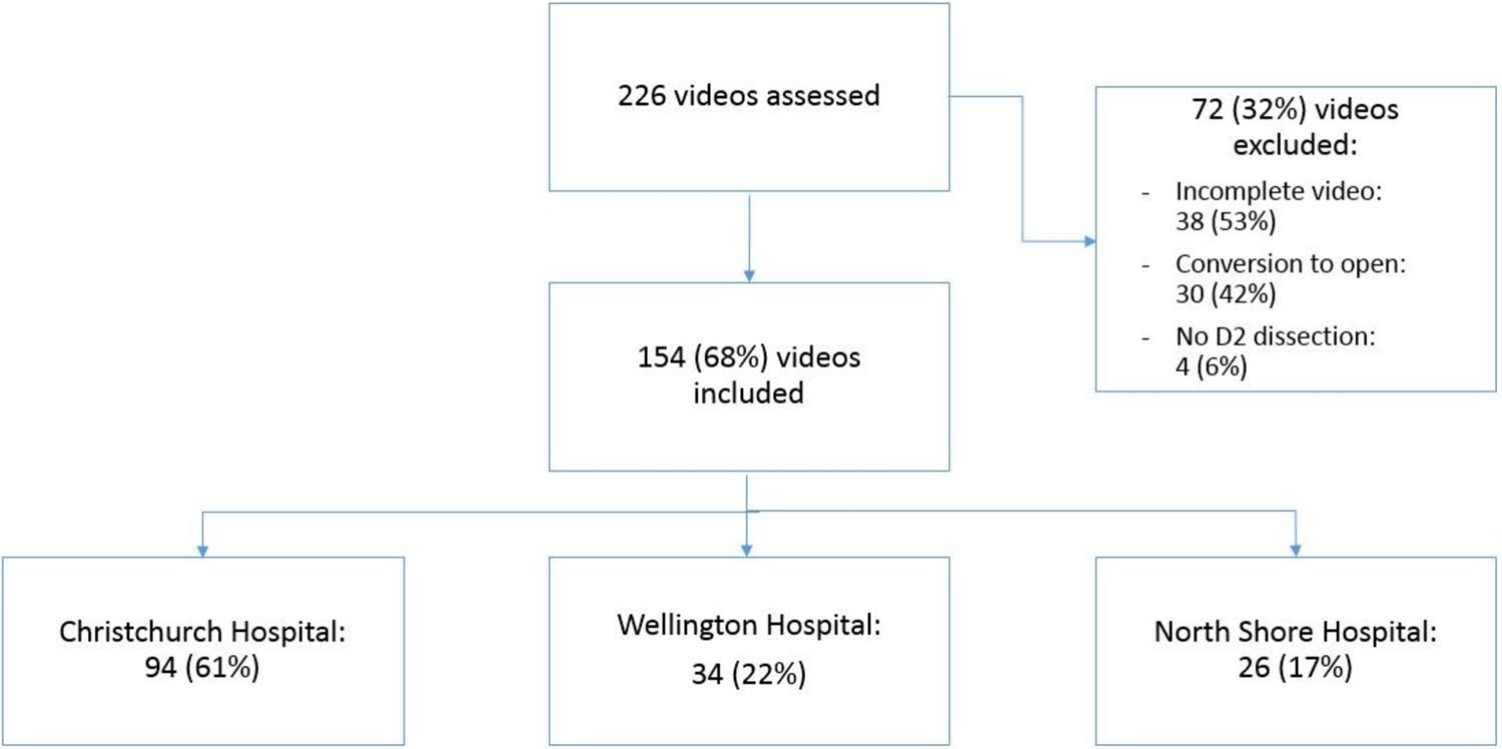
Flowchart study inclusion and exclusion

A total of 226 videos of colorectal resections were reviewed. In total, 154 patients were included between December 2020 and November 2023 from three institutions in New Zealand (Fig. 1). However, 72 patients were excluded; this was owing to incomplete video, conversion to open or vascular pedicle not taken with oncologic intentions (no D2 dissection).

The median age was 67 years (interquartile range (IQR) 60–75). In addition, 80 (51.9%) patients were female and 135 (87.7%) identified themselves ethnically as New Zealander/European.

In total, 80 (52%) patients underwent a left-sided resection (43 (54%) high anterior resection (HAR) and 37 (46%) low anterior resection (LAR)) and 74 (48%) patients underwent a right-sided resection (59 (80%) right hemicolectomy and 15 (20%) extended right hemicolectomy). Moreover, 135 (89%) operations were performed owing to a malignancy and 19 (12%) owing to benign pathology. Benign pathology included: tubovillous adenoma, five (26%) patients; Crohn’s disease, five (26%) patients; diverticular disease, seven (37%) patients; gynaecologic pathology, one (5%) patient; and lipoma-causing intussusception, one (5%) patient.

The median CAT score was 33 (IQR 32.5–35.5). The Kendall’s tau correlation test between the two assessing authors was 0.71.

**Fig. 2:**
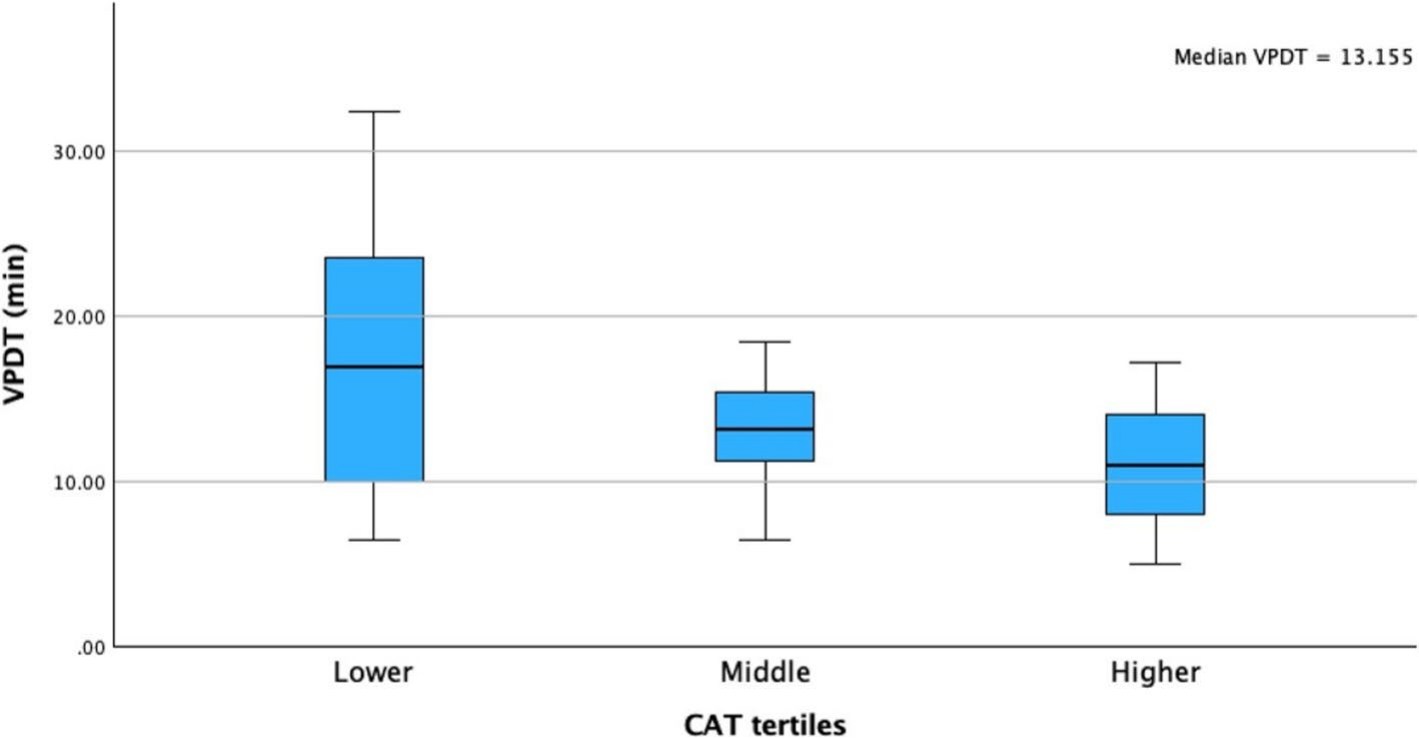
Median VPDT times (in minutes) divided into tertiles for patients who underwent a right hemicolectomy

**Fig. 3:**
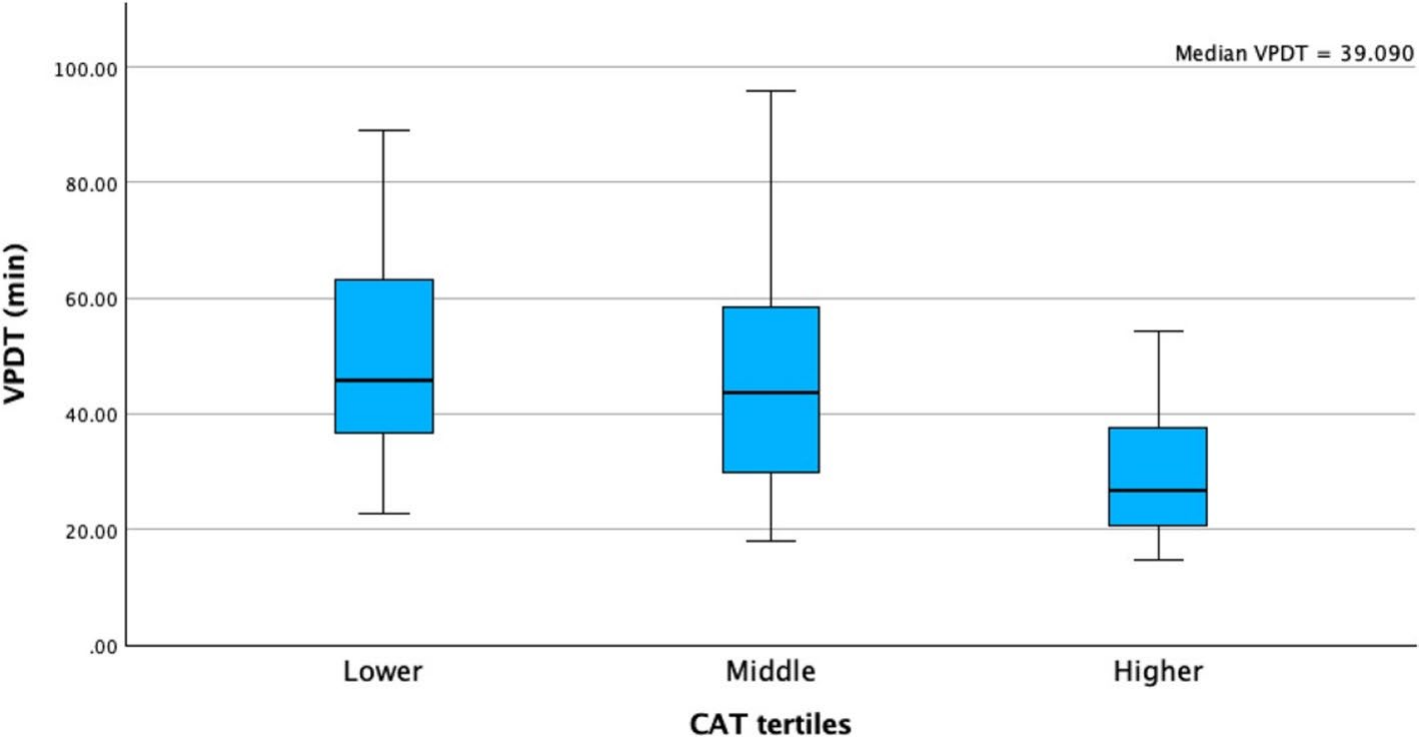
Median VPDT times (in minutes) divided into tertiles for patients who underwent an anterior resection

For patients undergoing a right hemicolectomy, the median VPDT in the lower tertile was 17 min (10–24), in the middle tertile 13 min (11–16) and in the higher tertile 11 min (8–14), *p* = 0.036 (Fig. 2). For patients undergoing a left-sided resection, the median VPDT in the lower tertile was 46 (36–65), in the middle tertile 44 (29–58) and in the higher tertile 27 (21–38), *p* =  < 0.001 (Fig. 3). Skin-to-skin operating time for right-sided resections was 144 min (115–169) for lower tertile, 120 min (100–150) for the middle tertile and 110 min (95–156) for the higher tertile, *p* = 0.102. For the left-sided resections, this was 252 min (214–291) for the lower tertile, 240 min (221–289) for the middle tertile and 208 min (175–245) for the higher tertile, *p* = 0.043. Ligation time for the right side was 16 min (8–21) in the lower tertile, 12 min (9–14) in the middle tertile and 9 min (6–12) in the higher tertile (*p* = 0.048). On the left side, this was 37 min (29–53) in the lower tertile, 37 min (27–50) in the middle tertile and 22 min (17–33) in the higher tertile (*p* = 0.019).

Patient, pathology and operation characteristics and their correlation to the CAT score are outlined in Table 1. Both median age and body mass index (BMI) were significantly different between the CAT tertile groups. In multiple regression analysis, only BMI remained statistically significantly different between the three CAT groups (*p* = 0.009). When the VPDT was divided into tertiles, none of the patient, pathology or operation characteristics were significantly different between the three groups.

**Table 1:** Patient, tumour and operation characteristics divided by CAT score outcome

	Lowest tertile CAT 24–33	Middle tertile CAT 33–35	Highest tertile CAT 35–43	*p* value
Patient characteristics				
Age (years; median (IQR))	70 (58–77)	71 (58–78)	63 (54–75)	0.039*
Gender				
Female (*n*, %)	27 (53)	26 (50)	27 (53)	0.942
Ethnicity				
NZ/European (*n*, %)	47 (92)	44 (85)	44 (86)	0.201
BMI (median (IQR))	29 (26–32)	27 (23–31)	25 (22–28)	0.023*
Tumour characteristics				
Pathology (*n*, %)				
Malignant	48 (94)	45 (87)	42 (82)	0.187
Neoadjuvant treatment *n*, %)				
Yes	6 (12)	11 (23)	4 (9)	0.121
T (*n*, %)				
≥ 3	29 (60)	27 (59)	22 (52)	0.725
*N* (*n*, %)				
+	17 (35)	14 (30)	15 (36)	0.983
M (*n*, %)				
+	0	1 (3)	0	0.336
Operation characteristics				
Operating surgeon (*n*, %)				
Consultant	9 (18)	16 (31)	11 (22)	0.415
Colorectal fellow	27 (53)	20 (39)	21 (41)	
General surgery trainee	15 (29)	16 (31)	19 (37)	
Operation (*n*, %)				
Right hemicolectomy	23 (46)	17 (33)	19 (38)	0.582
Extended right	5 (10)	5 (10)	5 (10)	
HAR	10 (20)	14 (27)	17 (34)	
LAR	12 (24)	16 (31)	9 (18)	
Mobilisation order (*n*, %)				
Ileocolic first	20 (39)	17 (33)	18 (35)	0.818
Hepatic flexure first	8 (16)	4 (8)	6 (12)	
IMA first	18 (35)	24 (46)	22 (43)	
IMV first	5 (10)	7 (14)	5 (10)	

*CAT* competency assessment tool, *IQR* interquartile range, *NZ* New Zealand, *BMI* body mass index, *T* tumour, *N* nodes, *M* metastases (as per the American Joint Committee on Cancer, TNM system), *HAR* high anterior resection, *LAR* low anterior resection, *IMA* inferior mesenteric artery, *IMV* inferior mesenteric vein

A *p* value of < 0.05 was considered statistically significant*

The occurrence of adverse outcomes, grouped between the CAT tertiles, is shown in Table 2. There were no R1 or R2 resections in this cohort.

**Table 2:** Outcomes divided by CAT score outcome

	Lowest tertile CAT 24–33	Middle tertile CAT 33–35	Highest tertile CAT 35–43	*p* value
Anastomotic leak (*n*, %))	1 (2)	2 (4)	1 (2)	0.785
CD > 3 complication (*n*, %)	3 (6)	6 (12)	4 (8)	0.577
Re-admission (*n*, %)	6 (12)	7 (14)	8 (16)	0.846
LOS (days; median (IQR))	5 (4–6)	6 (4–9)	5 (4–7)	0.818

*CAT* competency assessment tool, *CD* Clavian–Dindo, *IQR* interquartile range, *LOS* length of stay

A *p* value of < 0.05 was considered statistically significant*

## Discussion

This study shows that the VPDT is inversely related to the CAT score; the longer the dissection around the vascular pedicle takes, the lower the CAT score. The CAT score was only affected by BMI, showing that a higher BMI was associated with a poorer CAT score. No other patient, tumour or operative characteristics were associated with a poorer CAT score. There was no association between any of the patient, pathology or operation characteristics and the VPDT. Bleeding and tissue avulsion are scored in the CAT score negatively, this is more likely to occur in fatty mesenteries. Therefore, the authors think this explains the difference in the CAT score. A previous study found that increased BMI is associated with longer operative time [23]. In this study, operative time was inversely related to the CAT score; therefore, this can explain the relation between BMI and CAT score.

The CAT score was independently assessed by two of the authors. A correlation between the two CAT scores was performed and this showed a Kendall’s tau of 0.71; this is very strong, suggesting good correlation between the two assessors [22].

The VPDT was chosen between retraction of the pedicle to completion of the medial dissection and moving on to the next step of the operation. This was considered to be more consistent than the ligation time (from retraction to ligation), as some surgeons might choose to ligate the pedicle first and complete the dissection afterwards, and this is reflected in the comparison between median times per tertile. The end marker of ‘finishing the medial dissection of the operation’ is an obvious moment during laparoscopic surgery, as re-orientation of the camera to a different surgical field is needed to complete the subsequent part of the operation. For instance, on the right side, the surgeon visualises either the hepatic flexure or lateral attachments and on the left side, either the pelvis or splenic flexure may be visualised.

Operation duration or ‘skin-to-skin time’ has been associated with patient outcomes in a number of studies across multiple surgical specialties [24–26]. A recent study found that prolonged operative time not only was an independent risk factor for increased LOS in the hospital and in the intensive care unit but also reduced overall and disease-free survival in patients with colorectal cancer [26]. Skin-to-skin time can, however, be prolonged for a number of reasons, including: adhesiolysis, additional resections, operative logistics, etc. In this study, skin-to-skin time was not as significantly associated with the CAT score as opposed to the VPDT, and the authors believe this is because the VPDT is more consistent and less affected by patient and pathology characteristics.

Because the dissection around the vascular pedicle is a consistent and key step of the operation, evaluating and monitoring the skills of the surgeon at this particular step can be useful for surgical training and as a key performance indicator or textbook outcome parameter [27, 28]. Despite this promise, quantification of this metric is limited by the time taken to review the operative video. By utilising increasingly accurate artificial intelligence (AI)-driven operative phase detection models, this problem can be addressed [29, 30]. In practice, trainees would be able to record their operations and submit this for automated review with the output used to track their progress along the learning curve. The retrospective nature of this analysis ensures patients are protected from ethical dilemmas raised by real-time AI-driven clinical decision-making. Using clinically-derived metrics also ensures that AI technologies are adding value in clinical practice. A study in the same institutions in New Zealand is in progress, with the aim of automating the analysis of the VPDT using a three-dimensional convolutional neural network for video feature extraction and phase detection.

A limitation of this study was that it was not powered to evaluate the occurrence of adverse outcomes and correlate this to the CAT score or the VPDT. However, the CAT score is a validated score and a low score has been associated with overall poorer outcomes for the patient [12]. This study shows that the VPDT correlates with the CAT score; therefore, the authors believe that a longer VPDT is associated with an increased occurrence of adverse outcomes. A larger study will be undertaken in the same institutions in New Zealand to confirm this hypothesis. Another limitation is that all the different surgical approaches were included for the resections. The order in which the mobilisation was performed did not significantly differ between the CAT score group, nor was it significantly associated with VPDT. This might be because this study is underpowered to assess this. A recent study found that there is better interrater reliability for CAT score outcomes if the colorectal resection is performed in a standardised manner [31]. Lastly, for this study we used an abbreviated version of the CAT score, as our focus was only on the vascular pedicle dissection and the dissection. The anastomosis was deliberately left out in the assessment. The authors do not believe this altered the outcomes.

The CAT score was grouped into tertiles, which can be considered arbitrary. This is similar to previous studies that have used the CAT score for similar purposes [12, 32].

## Conclusions

This study showed that the VPDT correlated with a higher level of operative technical skill as quantified by the CAT score. Low CAT scores have been associated with worse post-operative outcomes. Although this study was underpowered to prove this for the VPDT, the correlation between the two is suggestive and can be confirmed in a larger cohort study. The VPDT is suitable for measurement using computer vision algorithms, which could allow for automated assessment of surgeons’ learning curve and skills.

## Data Availability

No datasets were generated or analysed during the current study.
